# Successful Subsequent Pregnancy in a Woman Receiving Eculizumab for Pregnancy-Associated Atypical Haemolytic Uraemic Syndrome

**DOI:** 10.1155/2019/2738723

**Published:** 2019-10-07

**Authors:** Samantha Bateman, Maleeka Ladhani, Shilpanjali Jesudason

**Affiliations:** ^1^Central and Northern Adelaide Renal and Transplantation Service (CNARTS), Royal Adelaide Hospital, Adelaide, Australia; ^2^Sydney School of Public Health, University of Sydney, Australia; ^3^School of Medicine, University of Adelaide, South Australia, Australia

## Abstract

Atypical haemolytic uraemic syndrome (aHUS) is a form of thrombotic microangiopathy precipitated by unopposed complement activation, the treatment of which has been revolutionised by the availability of the monoclonal anti-complement (C5) antibody, eculizumab. Historically, women with aHUS would be unable to achieve a successful pregnancy due to the severity of their renal disease and for the few who could conceive, recurrence of aHUS was a significant risk. In spite of this, parenthood remains a priority for many. Experience with eculizumab use in the management of aHUS during pregnancy is growing and with it comes a significant change in the course of the disease. We present the case of a 28-year-old woman diagnosed with severe aHUS in the first trimester of her first pregnancy. She received rescue therapy with eculizumab and had a return to normal renal function. While this pregnancy was lost, she strongly desired a family. We managed her through a subsequent pregnancy while receiving eculizumab. This pregnancy was uncomplicated and carried to term and she birthed a healthy 2500 g baby girl. The complexities of managing a pregnancy in a woman with a history of aHUS are vast but not insurmountable, as demonstrated by this case.

## 1. Introduction

Atypical haemolytic uraemic syndrome (aHUS) is a form of thrombotic microangiopathy (TMA) precipitated by unopposed complement activation. Until 2009 aHUS was managed similarly to thrombotic thrombocytopenic purpura (TTP) with plasma infusion and/or exchange, the efficacy of which was poor [1]. Over the last decade the treatment of aHUS has been revolutionised by the recognition of the underlying pathophysiology and the availability of the monoclonal anti-complement (C5) antibody, eculizumab. This treatment has improved outcomes for those with aHUS to the extent that return to normal or near normal renal function is a common, if not expected, outcome of treatment [1].

Women with a history of aHUS historically would be rendered unable to achieve a successful pregnancy due to the severity of their renal disease and for the few who could conceive, recurrence of aHUS during the pregnancy and peri-partum period remained a significant risk [2]. Parenthood remains a goal and priority for a woman with all forms of chronic kidney disease (CKD) [3] including aHUS. aHUS that occurs during or immediately post pregnancy, known as ‘Pregnancy associated aHUS' (P-aHUS) is associated with high maternal and foetal morbidity and mortality with more than 50% of woman progressing to end-stage kidney disease (ESKD) [2]. The complexities of managing a pregnancy in a woman with a history of P-aHUS, are vast but not insurmountable, as demonstrated by this case.

## 2. Case

**Figure 1:**
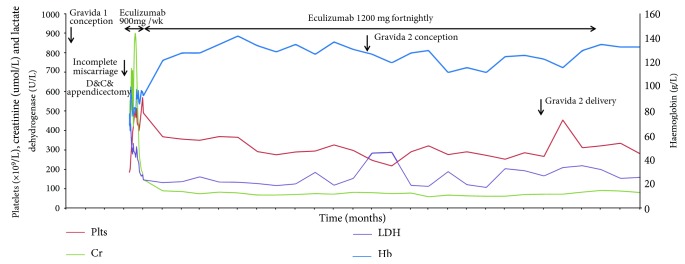
Trends of haemoglobin (Hb), platelets (Plts), creatinine (Cr), and lactate dehydrogenase (LDH) over time.

This report describes the case of a 28-year-old woman with no past comorbidity or relevant family history. Her first pregnancy (G1P0) was conceived via in-vitro fertilization with ongoing oestradiol therapy in the first trimester and suspected ovarian hyper-stimulation syndrome. Her baseline blood pressure was within normal limits. She presented with abdominal pain and per vaginal bleeding at 10-weeks gestation. She proceeded to have a dilation and curettage of an incomplete miscarriage, and also underwent a laparoscopic appendectomy for presumed appendicitis. On the first post-operative day she developed rapidly progressive, severe haemolytic anaemia with thrombocytopenia, liver function abnormalities, dialysis-requiring acute kidney injury (Figure 1), pancreatitis and pulmonary oedema requiring a brief period of ventilatory support. aHUS was diagnosed based on the clinical scenario and ADAMTS13 activity >70%. The patient was initially treated with plasmapheresis, but ultimately stabilized with urgent eculizumab therapy of 900 mg weekly for four weeks, with antibiotic coverage for meningococcal disease and subsequent vaccination. She made a rapid clinical recovery, with return to normal renal function within 2 weeks. She was Shiga-toxin negative. Subsequent genetic sequencing for C3, CD46, CFB, CFH, CFHR1-3 & 5, CFI, DGKE, MMACHC, PIGA, and THBD did not reveal any complement-related mutations or genetic variants associated with aHUS. Secondary forms of aHUS were considered but given the degree of clinical uncertainty and the severity of her presentation, eculizumab therapy was continued. She transitioned to a maintenance dose of 1.2 g fortnightly, with no evidence of further haemolysis and normal kidney function. In the context of her ongoing remission, the patient made it clear that she wished to pursue a further pregnancy. There was extensive counselling with obstetric nephrology and reproductive medicine experts regarding family planning including the uncertainties of IVF and pregnancy whilst receiving eculizumab.

**Figure 2:**
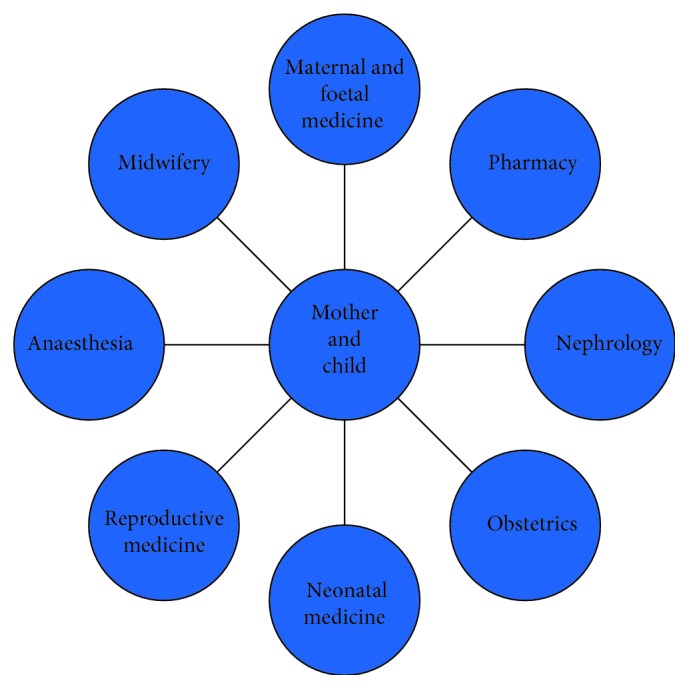
The integrated, multidisciplinary team involved in the provision of co-ordinated care of this high-risk pregnancy.

Fourteen months after her initial diagnosis, while still receiving eculizumab and in clinical remission with a serum creatinine of 80 *μ*mol/L, the patient underwent a successful low-oestrogen in-vitro fertilization process and live embryo implantation resulting in a successful pregnancy. The pregnancy was uncomplicated with no infection, haemolysis or renal dysfunction (Figure 1). Her urine remained bland without any detectable blood or protein and her blood pressure within normal range without the need for treatment. Haptoglobin, C3, and C4 were measured in each trimester and remained normal throughout the pregnancy. Fortnightly blood films were reviewed and did not show any schistocytes. C5 and free eculizumab levels were not available at our institution. She had close monitoring of foetal growth with involvement of maternal-foetal medicine and an expert multidisciplinary team (Figure 2). She was delivered at 37 weeks via elective caesarean section, due to patient preference. The healthy female infant had a birthweight of 2500 g, on the 14th centile. The patient remained well and received eculizumab throughout the pregnancy and post-partum period. As per Australian government access scheme guidelines she ceased eculizumab therapy 2 years after initiation, which was 8 weeks post-partum. At the time of writing, six months post-cessation of therapy there has been no sign of relapse. She is not planning a further pregnancy.

## 3. Discussion

The treatment of aHUS has been revolutionised by the availability of eculizumab. Pregnancy, which was once considered relatively contraindicated for women with history of aHUS, is now achievable. This case highlights a number of the complexities in managing women who have experienced P-aHUS and wish to have a subsequent pregnancy.

In 2010, Fakhouri et al. [2] identified and implicated alternative complement pathway dysregulation as the underlying pathophysiological aetiology of P-aHUS. Prior to this time, P-aHUS had been considered a secondary cause of aHUS which should, in turn resolve with resolution of the pregnancy. In that study of 100 women with a history of aHUS, they identified that 21% of cases were associated with pregnancy: 18 were de-novo and 3 were cases of relapsed aHUS during a subsequent pregnancy. The prevalence of complement abnormalities in P-aHUS was 86%, which is in fact higher than the rate of complement abnormalities (76%) in woman with nonpregnancy aHUS. This finding has been confirmed in several subsequent studies [4–6] which report rates of genetic complement abnormalities in woman with P-aHUS within the expected prevalent range of 27–59% of adults with nonpregnancy associated aHUS [1]. The importance of this recognition of the underlying pathophysiology of P-aHUS is magnified by the availability of a specific anti-complement therapy in eculizumab.

P-aHUS most commonly presents in the post-partum period, typically in the first week post-delivery [2]. Less frequently it may occur during the pregnancy, a tendency that has been noted to be more common in women with a personal history of aHUS [4], this is typically in the third trimester with only rare cases seen in early pregnancy [2]. The timing of P-aHUS is in contrast to other forms of TMA that may complicate pregnancy such as TTP and haemolysis with elevated liver enzymes and low platelets (HELLP), which more commonly occur earlier in the pregnancy; in the first and third trimesters respectively [7].

The mechanism by which pregnancy precipitates unopposed complement activation remains unclear. In normal pregnancy the placenta plays an important role in complement activation but is tightly regulated by two specific inhibitory proteins, CD59 and Decay Accelerating Factor, neither of which have been implicated in genetic pathophysiological mechanisms that lead to aHUS [4]. When the placenta is removed and these factors are no longer expressed this balance may be disrupted and unopposed activation triggered, which in part explains the predilection of P-aHUS to post-partum presentation. The timing in this case, at 10-weeks gestation, was unusual but may be explained by alterative triggers of complement activation namely the oestrogen hyper-stimulation, incomplete miscarriage and surgical intervention with dilation and curettage.

Mrs FG does not have a currently identifiable complement gene variant to explain her P-aHUS. The science around genetic testing for pathologically relevant variants in aHUS continues to evolve. Simplistically, missense variants in complement factor H, complement factor I and membrane cofactor protein result in impaired function of these inhibitory proteins, while pathogenic variants of C3 and complement factor B result in gain-of-function mutations, which in turn result in unregulated activation [1]. Novel and rare mutations have been identified but their pathological significance remains unclear including some variations that are more common in P-aHUS [1].

Given the historically devastating prognosis of aHUS there is very little evidence around the risk of relapse, maternal and foetal outcomes of women with a history of aHUS who proceed with pregnancy. Two recent studies [4, 8] have followed women with a history of aHUS through subsequent pregnancies and have reported variable outcomes. Servais et al. [8] followed three patients with a history of aHUS through five pregnancies, four of which were treated with eculizumab. They found increased rates of pre-term birth, growth retardation and pre-eclampsia but no recurrence of aHUS. It must however, be noted that all of these women had at least moderate CKD and hypertension, both of which are known risk factors for the above-mentioned complications.

In contrast, Gaggl et al. [5] followed 14 patients with a history of aHUS through 27 pregnancies, 7 of which were complicated by TMA. When extrapolated through the cohort this gives a rate of 6.94 episodes per 1000 weeks-at-risk for those with a known or unknown predilection to aHUS [5]. Three of the seven pregnancies complicated by recurrent aHUS appeared to be provoked by an additional known trigger; infection, bleeding and spontaneous abortion necessitating curettage. Seven of the 14 woman had a history specific for P-aHUS. Together they had 14 pregnancies, six of which were managed prospectively. Pregnancy outcomes within this sub-cohort, none of whom received prophylactic eculizumab, varied significantly. Rates of adverse foetal outcomes were 22 per 100 pregnancies where the woman had a history of aHUS, this increased to 70 per 100 pregnancies in those pregnancies that were complicated by P-aHUS. Term birth occurred in 70% of these pregnancies; 15% had spontaneous abortion before the 21^st^ week and 15% resulted in pre-term birth, half of which were still-births, with adverse foetal outcomes more common in those with pre-pregnancy renal impairment.

Given its relative rarity, the optimal management of P-aHUS remains unclear. Plasma exchange (PEX) is not effective with high rates (up to 50%) of progression to ESKD, similar to those who did not receive PEX [4]. Eculizumab however, has been shown to be similar in its efficacy in treating P-aHUS as nonpregnancy related aHUS. A Spanish registry study by Huerta et al. [6] found that the use of eculizumab in the management of P-aHUS improved renal survival at 24 months from 50% to 100% (*p* < 0.05), independent of the presence of a known genetic complement variation. The required duration of treatment remains unclear. Eculizumab was discontinued in 7 of 10 patients after a median of 10 months, subsequently 2 (29%) patients relapsed at 5 and 7 months post discontinuation and required further treatment [6].

The safety of eculizumab during pregnancy remains unknown but experience is growing and it is important to continue reporting cases. Pregnant women were excluded from the prospective HUS trials with eculizumab [4], however; studies of women with paroxysmal nocturnal haemoglobinuria treated with eculizumab during pregnancy have been encouraging with no increase in foetal adverse effects in this group. Eculizumab has been shown to cross the placenta but even when at therapeutic levels in the mother does not affect complement activation in the newborn, it is not known to be excreted in breast milk [9].

Given the paucity of experience with eculizumab in P-aHUS, an optimal dosing regimen is yet to be established. The efficacy of standard dosed eculizumab is thought to fall in pregnancy, demonstrated by an increase in CH50 levels [8]. This is hypothesised to be due to an increase in its metabolism in addition to the physiological changes of pregnancy causing an increased volume of distribution and effective dilution of the active drug, a phenomena which is most apparent in the later stages of pregnancy. Despite this relative dose reduction, in what is to our knowledge, the largest study of eculizumab use in P-aHUS [8], Servais et al. did not see any associated clinical manifestations of the residual complement activation at standard dosing. C5 inhibition in this same cohort remained inadequate despite increases in both dose and administration frequency of eculizumab and is thought to potentially be due to increased C5 production during pregnancy [8].

Monitoring of complement blockade during pregnancy, by way of C5 and free eculizumab levels with a dose increase in those women with incomplete C5 inhibition (from the recommended 1200 mg) to 1500 mg fortnightly has been trialled [8]. However, the dose–response curve of eculizumab is known to be nonlinear, which means to achieve saturation of complement blockade a very high dose of eculizumab may be required. What is not known is the degree of complement blockade required to prevent clinical manifestations of unopposed complement activation, particularly in the pregnant state in order to prevent a relapse of aHUS [8].

Despite the risks and the relative unknowns, pregnancy remains a priority for many women with all forms of CKD, including those with aHUS. Feelings of fear, grief, and anxiety for woman surrounding the failure to fulfil social norms and forgoing parenthood are contrasted by those related to disease exacerbation, birth defects and even death [3]. Pre-pregnancy counselling with shared decision-making regarding family planning that specifically takes into consideration the woman's experiences, values and priorities is a vital part of holistic medical care. Furthermore, multidisciplinary pregnancy care with a patient-specific team has been shown to optimise the management of pregnancies in woman with CKD [3].

## 4. Conclusion

This case describes a successful pregnancy outcome in a woman with a history of foetal loss and P-aHUS, with the use of prophylactic eculizumab before, during and after pregnancy. Eculizumab appears to be safe and effective in pregnancy however the pharmacokinetics and long-term sequelae of its use remain unknown. Pre-pregnancy counselling with shared decision-making along with integrated multidisciplinary pregnancy care is essential in the management of women with aHUS who wish to, or successfully become pregnant in order to achieve the best outcomes for both mother and child.

## Additional Points

*Teaching points:* Women with a history of aHUS, including P-aHUS are able to achieve a successful pregnancy. Eculizumab appears to be safe and effective in pregnancy however the long-term sequelae of its use remain unknown.Pre-pregnancy counselling for women with a history of aHUS is essential. Integrated care, through a multidisciplinary team approach, works to achieve best outcomes for high risk pregnancies including those with aHUS.
